# Don’t Lose Sleep Over Neurodegeneration-It Helps Clear Amyloid Beta

**DOI:** 10.3389/fneur.2013.00206

**Published:** 2013-12-19

**Authors:** John McKinley, Allan McCarthy, Timothy Lynch

**Affiliations:** ^1^Department of Neurology, The Dublin Neurological Institute at The Mater Misericordiae University Hospital, Dublin, Ireland; ^2^Dublin Academic Medical Centre, Dublin Neurological Institute, Dublin, Ireland

**Keywords:** sleep, amyloid, neurodegeneration, cerebrospinal fluid

Xie et al. in their article entitled “sleep drives metabolite clearance from the adult brain” in Science (342: 373–377) (1), provide an important insight into the potential physiology behind the restorative and homeostatic nature of sleep, a basic requirement for normal brain function. This study, utilizing a mouse model explores the clearance of peptides such as amyloid beta, which is implicated in the pathogenesis of Alzheimer’s disease (2) and may offer a platform for the investigation of other forms of neurodegeneration associated with other putative neurotoxic proteins.

Key to understanding their paradigm is an exploration of the differences between physiological clearance of cellular waste from the central nervous system and peripheral tissues. Systemic waste interstitial proteins are transported via lymph vessels and the systemic circulation to the liver for degradation (1). The brain lacks this mechanism and relies on the recirculation of cerebrospinal fluid, which acquires waste proteins as it flows through the interstitial spaces surrounding brain cells (2) before interfacing with the systemic circulation at the arachnoid granulations. This convective mechanism constitutes the glymphatic system and relies on aquaporin 4 (AQP4) water channels for effective clearance of waste solute. The authors highlight that many of the putative proteins associated with neurodegeneration are found in the interstitial fluid surrounding brain cells and that knocking out AQP4 channels for example reduces the clearance of amyloid beta (Aβ) by 65% (1).

Xie et al. used a mouse model, with an implanted cannula for delivery of a fluorescent tracer via the cisterna magna into the CSF, to observe real-time movement of CSF *in vivo* using two-photon imaging (1). This technique allowed the influx of CSF into the murine cerebral cortex to be monitored “real-time” in three states; sleep, awake, and anesthetized.

Electrocorticography (ECoG) and electromyography (EMG) determined sleep-wake status, with a sleep state practically defined as an increased power of slow waves and an awake state associated with a relative reduction in slow waves and increase in power of fast waves (1).

On entering sleep there was a “robust influx” of fluorescent CSF tracer along the periarterial spaces, subpial region, and into the brain parenchyma (1). Sleep was confirmed by the presence of a high power of slow waves on the ECoG.

On awakening, CSF tracer influx reduced dramatically by 95% (1). The state of wakefulness was confirmed by a change in the ECoG (1).

These results were replicated in a cohort of mice, examined firstly whilst awake. The intracisternal infusion of fluorescent CSF tracer was associated with only minimal influx into the brain. Then on induction of anesthesia there was a rapid influx of CSF tracer along the periarterial spaces into the brain parenchyma, similar to naturally sleeping mice (1).

They postulate that CSF influx into brain parenchyma is not simply a function of arterial pulsation and blood pressure but rather the interstitial space volume is dynamic and contracts during wakefulness and expands during sleep (1).

To test this, they used the real-time iontophoretic tetramethylammonium (TMA^+^) method whereby TMA^+^ was delivered via an iontophoresis microelectrode in the vicinity of a TMA^+^-sensitive microelectrode stationed approximately 150 μm away in the interstitial space. In the paradigm a small interstitial space results in reduced TMA^+^ dilution (as volume of interstitial space is reduced) after delivery and thus higher levels of residual TMA^+^ are detected by the sensing electrode (1) and vice versa. Essentially they confirmed that the interstitial space volume fraction was increased by over 60% in anesthetized or sleeping mice compared to when they were awake (1).

Knowing that approximately 65% of exogenously delivered Aβ is cleared by the glymphatic system they decided to explore whether interstitial Aβ is cleared most efficiently during sleep (1). Radiolabeled ^125^I-Aβ_1–40_ was injected into the cortex of awake mice, sleeping mice, and anesthetized mice (1). Brains were examined for levels of residual ^125^I-Aβ_1–40_ between 10 and 240 min after injection and they established that Aβ was cleared twice as fast in sleeping and anesthetized mice compared to awake mice. Furthermore, to account for additional receptor-mediated clearance of Aβ across the blood brain barrier, an inert ^14^C-inulin tracer was shown to be cleared more than twice as fast in sleeping and anesthetized mice compared to awake mice (1).

There are two possible contributory factors. Either interstitial space volume is influenced by circadian rhythm or it is a function of the sleep-wake state of the individual (1). The authors assert that it is sleep-wake state rather than circadian rhythm that is fundamental as induction of anesthesia at a time usually associated with natural wakefulness results in identical interstitial space volume and Aβ clearance to that found in natural sleep (1). Based on this premise, they postulate that the noradrenergic (NA) system controls the interstitial space volume by driving arousal. Thus, they hypothesized that blockade of NA during wakefulness would result in an increase in CSF influx into the brain. Xie et al. infused an adrenergic antagonist into the cisterna magna during wakefulness whilst measuring CSF influx using their original fluorescent CSF tracer method and found that the infusion of adrenergic antagonists increased CSF influx similar to that seen in the sleeping state (1). Using the TMA^+^ iontophoresis method they further confirmed that NA antagonism increased the brain interstitial volume in a similar fashion to sleep and anesthesia (1). The four principal experiments by Xie et al. (summarized in Table 1) have elucidated how cortical CSF influx and interstitial space volume are influenced by arousal state and how pharmacological intervention in the form of anesthesia or adrenergic antagonism can modify these parameters.

**Table 1:** **Summary of four principal experimental methodologies, results, and implications**.

Experiment	Methods	Results	Implications
Real-time observation of CSF influx to cerebral cortex	Infusion of fluoroscopic CSF tracer via a cannula implanted into the cisterna magna. Imaging *in vivo* using two-photon imaging in three states of arousal (awake, asleep, and under anesthesia)	When asleep: “robust influx” of fluorescent CSF tracer along the periarterial spaces, subpial regions, and parenchyma	Interstitial space volume is dynamic and a function of the state of arousal whether that be physiological states such as sleep or wakefulness or artificially induced states such as anesthesia
		When awake: CSF influx reduced “dramatically” by 95%	
		When anesthetized: on induction there was a rapid influx of CSF similar to the sleeping state	
Assessment of interstitial space volume during different states of arousal (sleep, wakefulness, or anesthesia)	Iontophoretic Tetramethylammonium method (TMA^+^): TMA^+^ delivered to cortex via an iontophoresis microelectrode situated 150 μm from a TMA^+^ sensitive microelectrode located in the interstitial space	Interstitial space volume fraction increased by over 60% during the sleeping or anesthetized state	Confirms that interstitial space volume is dynamic and influenced by state of arousal
Assessment of Aβ clearance during different states of arousal (sleep, wakefulness, or anesthesia)	Radiolabeled ^125^I-Aβ_1–40_ injected into the cerebral cortex. Brains harvested between 10 and 240 min after injection and residual ^125^I-Aβ_1–40_ measured	Aβ cleared twofold faster in sleeping/anesthetized mice compared to awake mice	Suggests that glymphatic efflux of waste interstitial proteins is responsive to changes in arousal state (both physiological states such as sleep and wakefulness and induced states such as anesthesia)
Determining if interstitial space volume is influenced by arousal state or circadian rhythm	Based on the premise that wakefulness is driven by noradrenergic tone, glymphatic tracer influx was assessed (after the local administration of adrenergic antagonist) using the fluoroscopic tracer method outlined above. Furthermore, interstitial space volume was assessed in response to noradrenergic antagonist using the iontophoretic TMA^+^ method outlined above	Adrenergic antagonist induced an increase in CSF tracer influx “more comparable” to that seen in the sleeping/anesthetized state than that seen during wakefulness. Adrenergic antagonist resulted in significant increase in interstitial space volume fraction	Adrenergic tone has a role in modulating arousal state and the volume of the interstitial space

Why do we need to sleep? The authors postulate that sleep is the homeostatic method responsible for the clearance of toxic metabolic byproducts accumulated during wakefulness. This study by Xie et al. raises many questions in the study of neurodegeneration. Could NA antagonists impact on the accumulation of putative toxic proteins in Alzheimer’s disease and other forms of neurodegeneration? Is rapid eye movement (REM) sleep disorder merely a manifestation of alpha synucleinopathies or could it be implicated in pathogenesis? Why do some patients with Parkinson’s disease derive sleep benefit? What causes fluctuating arousal in dementia with Lewy bodies (DLB)? Does sleep-wake state influence volumetric analysis in MRI? Given the invasive nature of the experimental methodologies used they could not be reproduced in humans in their current format, however they could provide a platform for the investigation of clearance of other neurotoxic proteins involved in neurodegeneration, using a Parkinson’s disease mouse model for example. Furthermore, they may be useful for investigation of potential drugs to promote the clearance of neurotoxic proteins, whether they be currently used adrenergic antagonist drugs or novel agents. When we try to encourage a good nights sleep for our patients perhaps we are doing them more good than we thought.
